# Exploring the Physiological and Psychological Effects of Group Chanting in Australia: Reduced Stress, Cortisol and Enhanced Social Connection

**DOI:** 10.1007/s10943-023-01967-5

**Published:** 2023-12-13

**Authors:** Gemma Perry, Vince Polito, William Forde Thompson

**Affiliations:** 1 School of Psychological Sciences, Macquarie University, Sydney, 2109 Australia; 2 Faculty of Society and Design, Bond University, Gold Coast, 4229 Australia

**Keywords:** Cortisol, Stress, Anxiety, Loneliness, Social connection, Chanting, Music, Mantra, Singing, Altrusim

## Abstract

Chanting, an ancient ritual practiced in diverse cultures and traditions worldwide, has typically been employed for meditation, healing, self-awareness, and psychological growth. However, there is little understanding of the physiological and psychological benefits of chanting, and how vocalization might contribute to such effects. This study aimed to determine whether 12-minutes of group chanting, through vocal or silent repetition of the sound “om,“ would reduce stress and anxiety, while increasing feelings of social connection, and whether vocal chanting would yield stronger effects. Thirty-four participants were randomly assigned to vocal or silent group chanting conditions. Saliva samples were collected before and after chanting to assess cortisol levels, while self-report measures included the *State Trait Anxiety Inventory* and the *Adapted Self-Report Altruism Scale* (including additional items on cross-cultural altruism). Following chanting, participants also provided a written description of their experiences. Both vocal and silent chanting resulted in significant decreases in cortisol levels and self-reported anxiety. The reduction in cortisol was similar for vocal and silent chanting, but self-reported anxiety decreased more following vocal chanting. Altruism scores increased following both vocal and silent chanting. However, there was no evidence of altruistic tendencies extending toward people from a culture other than one’s own. Results are discussed in relation to the phenomenology of chanting, and to current theory and evidence on the physiological and psychological effects of chanting and singing.

High stress, anxiety, social isolation, and loneliness have emerged as significant contributors to the development of mental health disorders, cardiovascular disease, and increased mortality rates (Hawthorne, 2008; Miller & Raison, 2016). Additionally, stress, anxiety and loneliness rose during the COVID-19 pandemic, forcing people to isolate and preventing them from maintaining their regular social support (Lee et al., 2020; Okruszek et al., 2020). Despite the need for effective interventions, current treatments have limited efficacy. For instance, pharmacological treatments targeting stress may be accompanied by adverse side effects that outweigh benefits, and many individuals report limited or no benefits from conventional antidepressant therapies (Rush et al., 2006). Therefore, an exploration of alternative interventions is needed to effectively manage negative consequences of stress, anxiety and social isolation.

In addition to medical and psychological interventions, religious/spiritual interventions are also commonly utilized and have benefits. In particular, chanting may confer physiological and psychosocial benefits and has recently garnered increased scientific interest. Studies by Perry et al. (2016) and Simpson et al. (2021) suggest that chanting can effectively reduce stress and enhance feelings of social connection. However, additional research is needed to validate these findings and elucidate the underlying mechanisms by which chanting elicits benefits. Chanting, characterized by rhythmic and repetitive singing or speaking, holds deep roots in diverse cultures, religions, and traditions (Perry & Polito, 2021). Perry et al. (2021) provided a comprehensive overview of its historical significance and widespread use as a tool for promoting health, facilitating spiritual development, fostering social connection, and serving as a form of worship. Given its rich heritage, chanting emerges as a potentially effective method for strengthening social bonds and alleviating anxiety.

Despite a wide range of chanting practices across cultures and traditions, there are common characteristics of chanting that may be associated with health benefits. Firstly, chanting typically involves rhythmic and repetitive singing, speaking or mental repetition of vocalized sounds and phrases that may facilitate slowed and rhythmic breathing (Bernardi et al., 2001). Second, chanting involves focused attention on the chosen sound or phrase that is repeated (vocally or silently) which may enhance focus and reduce ruminative thoughts or mind wandering (Bormann et al., 2014; Perry et al., 2022). If done in groups, chanting also involves synchrony with other members of the group through coordinated vocalisations, movement or breathing, known to enhance perspective-taking and connection to others (Novembre & Keller, 2014; Tarr et al., 2014). These features of chanting may contribute to its impact on positive mood, increased social connection, decreased stress and the alleviation of depressive symptoms (Oman & Bormann, 2015; Perry et al., 2021; Wolf & Abell, 2003).

## Physiological Stress

Chanting is strongly associated with feelings of relaxation, induced by a range of mechanisms including slowed breathing (Benson et al., 1974; Bernardi et al., 2000). Rhythmic, repetitive, and slow breathing during chanting engages the parasympathetic nervous system, potentially reducing physiological stress (Brown et al., 2013). In a study by Bernardi et al. (2001b), participants who engaged in 10 min of vocal chanting, whether a traditional Roman Catholic prayer (*the Ave Maria*) or a Buddhist mantra (*Om Mane Padme Hum*), exhibited a slowed breathing rate of 6 respirations per minute, a rate known to have positive effects on cardiovascular functioning (Tomasino et al., 2013). Apart from respiratory rate, there is limited understanding of the biological markers associated with psychological impacts of chanting.

One biological marker that may be associated with chanting is cortisol, a vital steroid hormone responsible for balancing healthy bodily functions such as regulating the stress response, metabolism, immune function, wound healing and bone formation (Li et al., 2013). Research has established an association between stress and cortisol, with prolonged exposure to high levels of cortisol associated with a range of health problems, including cardiovascular, metabolic, and digestive disorders (Selye, 1970). Moreover, excessive cortisol levels are associated with chronic stress, panic, depression, and post-traumatic stress disorder (PTSD; Brown et al., 2004; Fancourt et al., 2016). Therefore, cortisol serves as a reliable indicator of the stress response, with higher levels indicating more severe stress compared to lower levels (Strazdins et al., 2005).

A range of methods have been adopted to examine the impact of music on cortisol (Kreutz, 2014; Kreutz et al., 2004). Reduced stress and cortisol levels have been observed following various music, dance and singing interventions (Beck et al., 2000; Fancourt et al., 2016; Fancourt & Perkins, 2018; Sheppard & Broughton, 2020). Further, listening to music (compared to silence) has been found to prevent increases in cortisol levels in response to a stressor (Khalfa et al., 2003). Kuhn (2002) found that engaging in 30 min of singing and music-playing reduced cortisol more than solely listening to music. However, research has also indicated that listening to music alone can lower physiological stress (Kreutz et al., 2004). For example, Fukui and Toyoshima (2013) demonstrated that just 5 min of listening to chill-inducing music (defined as a powerful emotional response induced by music) led to decreased cortisol levels in saliva. Notably, cortisol reduction was observed regardless of whether participants listened to preferred or non-preferred music. Conversely, Vanderark and Ely (1993) found non-preferred music increased cortisol levels. On balance, these findings suggest that both singing and listening to music, especially preferred music, can lead to reduced physiological stress.

Research also suggests that cortisol is reduced by meditation techniques (Pascoe et al., 2017; Sanada et al., 2016), and mindfulness programs (Matousek et al., 2010). Research on chanting meditation has considered silent chanting techniques (Walton et al., 2005); employed longitudinal designs of 4 weeks – 4 months (Klimes-Dougan et al., 2020; MacLean et al., 1997); and considered specific populations such as postmenopausal women (Walton et al., 2004). One study revealed that nursing professionals (with moderate to severe stress levels) chanting the “Hare Krishna” Mahamantra (“Hare Rama Hare Rama, Rama Rama Hare Hare; Hare Krishna Hare Krishna, Krishna Krishna Hare Hare”) for 20 min daily for 45 days exhibited reduced cortisol levels, when compared to a (non-intervention) control group (Niva et al., 2021). However, the immediate effects of vocal chanting on cortisol in healthy populations are unknown. The current study aimed to address this gap by comparing silent chanting to vocal chanting and examining their immediate effects on physiological stress in healthy adults.

## Anxiety

As well as impacting physiological stress, chanting may also help to reduce anxiety through cognitive processes such as focused attention, which can enhance emotional and cognitive control through disengagement from automatic thought (Chiesa, 2013; Lutz et al., 2008; Sauer et al., 2013). These effects, in turn, have the potential to mitigate negative mental states associated with depression and low mood (Killingsworth & Gilbert, 2010; Malviya et al., 2022a, b). In a randomized controlled trial involving 146 veterans with PTSD, Bormann et al. (2014) found that a 6-week silent chanting program led to reduced PTSD symptoms and increased mindful attention compared to veterans receiving standard treatment. Similarly, Wolf and Abell (2003) reported that vocal chanting for 20 min daily over a 28-day period reduced stress and depressive symptoms. These studies demonstrate the efficacy of chanting in reducing psychological stress over a 4-6-week duration. However, stress management often requires rapid solutions, and the immediate effects of chanting on stress and anxiety are still unknown.

## Social Connection

Other benefits of chanting may include enhanced social connection. Group synchronisation is thought to be an important means of promoting cooperation and feelings of connection to others (Kniffin et al., 2016; Kokal et al., 2011; Tarr et al., 2014; Wiltermuth & Heath, 2009). Synchronous musical activities and rituals have been shown to enhance social connection, well-being, group problem-solving, and altruistic behaviour (Broughton, 2021; Dingle et al., 2021; Greenberg et al., 2021; Hobson et al., 2018; Kirschner Sebastian & Tomasello, 2010; Kniffin et al., 2016; Sheppard & Broughton, 2020). Therefore, group chanting, being a synchronous and musical activity, is likely to promote feelings of well-being and altruistic tendencies. Chanting in a group setting may offer advantages over chanting in isolation by enhancing feelings of social connection (Perry et al., 2016; Simpson et al., 2021). Altruism is a core principle that emphasises connection to others, characterised by voluntarily acting to benefit a social group with the absence of reward or personal gratification (Fehr & Fischbacher, 2003). Higher altruism is associated with pro-social behaviour and low altruism is associated with anti-social behaviour (Fehr & Fischbacher, 2003; Gerdes et al., 2011). The capacity of synchronous behaviour to promote altruism was demonstrated in a study by Kokal et al. (2011) who found that participants were more likely to help an experimenter following the study if they participated in synchronous drumming compared with asynchronous drumming. Additionally, Valdesolo et al. (2010) demonstrated coordinated action positively influenced cooperation and the pursuit of shared goals. In a randomized control study involving 92 participants, synchronous rocking in chairs improved joint-action task abilities. These findings collectively suggest that chanting in groups is likely to enhance social connection and altruistic tendencies given its synchronous nature.

It is also possible that chanting in a group will lead to feelings of altruism toward cultures other than one’s own. The most influential theory of positive intercultural relations is the ‘contact hypothesis’ suggesting that positive contact between individuals from various backgrounds should improve intercultural relations (Allport et al., 1954; Nesdale & Todd, 2000). Further, activities that involve group synchrony can lead to feelings that there are few or no boundaries between oneself and others, whereby high attentional focus and emphasis on unity of the group generates a sense of social cohesion (Wiltermuth, 2008). Chanting is a synchronous activity that may facilitate such a social connection through the interaction with others but it is unknown whether chanting might increase feelings of altruism toward cultures other than one’s own.

## Vocal vs. Silent Chanting

While the impact of the immediate effects of chanting on social connection remain under explored, there is also little understanding of the distinct effects of vocal and silent chanting on stress and social connection, with existing literature focussed on silent chanting. Vocal chanting may offer additional advantages over silent chanting owing to characteristics inherent in the vocalization process. Firstly, vocal chanting involves explicit sound production. Explicit vocalization has the potential to enhance social connection, because when individuals chant together vocally, they engage in a shared vocal activity, which can promote a sense of unity, belongingness, and cooperation. The act of vocalizing together in a group setting fosters a collective experience and can create a powerful sense of togetherness and solidarity, enhancing social relationships and reducing feelings of isolation (Boster et al., 2021; Pearce et al., 2015). Vocal chanting also involves synchronization to coordinated rhythmic patterns. When individuals chant in unison, their voices synchronize, and group synchrony has been linked to increased cooperation, empathy, and prosocial behaviors among individuals. By chanting in synchrony, individuals experience a shared temporal structure that promotes unity and cooperation, strengthening social connections and reducing stress (Perry et al., 2016; Simpson et al., 2021). Silent chanting, though lacking explicit vocalization and synchronization, can still offer benefits through distinct, but overlapping, mechanisms. Silent chanting relies on focused attention and repetition of sound imagery. When individuals engage in silent chanting, they direct their attention inward, focusing on the imagined repetition of sounds, ideas, words, or mantras. This focused attention cultivates a meditative state, promoting relaxation, mindfulness, and a sense of inner calm. The repetitive nature of silent chanting can induce a state of deep concentration and mental absorption, allowing individuals to enter a state of tranquillity and heightened awareness (Bormann et al., 2014; Perry et al., 2022).

The explicit vocalization in vocal chanting may also reduce stress more than silent chanting for physiological reasons, as the vocalization of sounds may directly impact respiration. This change in respiration may lead to slowed breathing and activation of the parasympathetic nervous system associated with relaxation (Jerath et al., 2006). While silent chanting incorporates elements such as focused attention that may assist in stress reduction, it is unlikely to have the same explicit impact on respiration as vocal chanting, and thus, less impact on reducing stress. However, merely listening to choral music has been found to decrease levels of cortisol, suggesting music engagement in the absence of explicit vocalization may still reduce physiological stress (Kreutz et al., 2004; Kuhn, 2002). Therefore, it is unknown whether vocal chanting will reduce cortisol more than silent chanting.

Vocal chanting may also promote stronger effects on altruism due to its capacity for creating high levels of group synchrony, whereas silent chanting lacks the explicit auditory-motor feedback required for synchronous behavior. The joint action task of vocalizing sounds in a group during vocal chanting is likely to contribute to a stronger effect on altruism compared to silent chanting. However, further research is needed to examine and compare specific effects of vocal and silent chanting on stress reduction and social connection.

## The Current Study

The aim of the current study was to investigate the effects of vocal and silent chanting on stress, anxiety and altruism. It was hypothesized that both vocal and silent chanting would lead to decreased stress and anxiety as well as increased altruism. However, it was anticipated that the effects would be more pronounced for vocal chanting. The study employed physiological markers of stress, self-reported anxiety, and a self-report measure of altruism. Additionally, an exploration of the subjective experiences associated with vocal and silent chanting was conducted. This study contributes to an understanding of potential benefits of chanting while exploring possible distinctions between vocal and silent chanting in terms of stress reduction, altruism, and subjective experiences.

## Method

### Participants

A total of 41 participants took part in the study: Two were excluded for response bias, assigning the same response on the Likert scale regardless of context (Paulhus, 1991). Specifically, these two participants selected a rating of 4 on every question in the *State Trait Anxiety Inventory*. This measure includes positively and negatively valanced items, so this pattern of responding is unlikely to reflect valid responses. Five additional participants were excluded for not providing a saliva sample.

Participants were randomly assigned to a vocal or silent group chanting condition. The final analyses were conducted on data obtained from 16 participants in the vocal condition and 18 participants in the silent condition (n = 34 total). There were 23 females and 11 males, ranging in age from 18 to 49 years (*M* = 21.79, *SD* = 7.17). Participants were recruited through the Macquarie University Psychology Participant Pool and were offered course credit in exchange for participating in the study. Ethics was approved by the Macquarie University Ethics committee and each participant provided consent.

### Materials

#### Chanting Recording

The experimental phase of the study involved vocally chanting with (vocal group) or listening to (silent group) a recording for 12 min. The recording is of a male voice that is chanting the sound of “om” for lengths of 10 s and without any instrumentation. This recording was chosen as it had similar timing to a previous chanting experiment which found two different chants from different traditions slowed breathing to 6 respirations per minute (Bernardi et al., 2001). The recording also served as a means of standardising the experimental conditions in terms of timing and sound between the groups. This recording is available on YouTube (Aggarwal, 2012).

#### Cortisol Measure (Before and After)

Salivary cortisol reflects levels of stress in the body and the collection of cortisol from saliva is non-invasive (Twal et al., 2016). Therefore, salivary cortisol was used as a measure of physiological stress and collected via the passive drool saliva collection aid supplied by Salimetrics. Participants were asked to allow saliva to pool in the mouth, tilt their head forward and then gently force saliva through a plastic mouth piece into a small vial and to fill as far as 1ml. Reliability of this method of saliva collection has been found to be good when compared with other collection methods (Strazdins et al., 2005). Samples were frozen at -20 °C within 2 h of collection until being sent for analysis. The samples were assessed using a commercially available ELISA assay (Salimetrics, USA) according to manufacturer’s instructions. Lower cortisol in the saliva indicates lower levels of stress.

#### The State Trait Anxiety Inventory (Before and After)

Self-reported anxiety was assessed using the State Trait Anxiety Inventory (STAI; Spielberger, 1983). Although this test consists of two self-report scales, the current study utilized the state anxiety scale (STAI-S), which was expected to change following the chanting intervention. The STAI-S consists of 20-items that measure temporary emotional state, asking participants to indicate how they are feeling in the present moment (e.g., “*I am worried*”). Each item is rated on a 4-point scale ranging from 1 (*not at all*) to 4 (*very much so*). Scores range from 20 being the lowest and 80 being the highest with higher scores indicating higher state and trait anxiety. The STAI-S has demonstrated good internal consistency (Cronbach’s α = 0.95) and test-retest reliability is suitably low (*r = .33*), appropriate for measuring transitory states (Spielberger, 1983). The current study had acceptable internal consistency before (Cronbach’s α = 0.71) and after (Cronbach’s α = 0.75).

#### Adapted Self-Report Altruism Scale (Before and After)

Altruism was measured with the Self-Report Altruism Scale (SRA; Rushton et al., 1981) consisting of 14 items where participants rated the frequency of engaging in altruistic behaviours (e.g., “*I would offer my seat on a train or bus to someone who was standing*.”). Each item is rated on a 5-point scale ranging from 1 (*never*) to 5 (*very often*). The total scale score is the sum of all items (ranging from 14 to 70). Psychometric properties of this scale are robust: it has been compared to an omnibus personality inventory and discriminant validity was found to be good (Rushton et al., 1981). Moreover, a low correlation between social desirability and the SRA (r = .05) indicates that scores on this measure are not influenced by socially desirable responding. The current study had good internal consistency before (Cronbach’s α = 0.85) and after the chanting intervention (Cronbach’s α = 0.87).

An additional six questions were included to assess whether altruism was also experienced toward other cultures following the intervention. These questions were developed for the current study and added to the SRA. These were more detailed questions about the culture of the person that participants were reporting to be altruistic towards. The 6 questions are as follows: “*I would help someone in the street regardless of their culture; I would donate money to an overseas charity; I would go out of my way to help someone from another culture; I would help a tourist in the street with directions; I would help someone from another country that was not familiar with how to purchase a ticket for something; I would go out of my way to help someone new from overseas feel part of the ‘team’ at work or in a group.”* Each item is rated on a 5-point scale ranging from 1 (*never*) to 5 (*very often*). The total score is the sum of all item scores (ranging from 6 to 30). The current study had excellent internal consistency before (Cronbach’s α = 0.90) and good internal consistency after the chanting intervention (Cronbach’s α= 0.83).

#### Chanting Reflections Task

To understand the phenomenology of chanting, participants were asked to write down their experiences, including thoughts, feelings or body sensations that occurred during or after chanting either silently or vocally. They were provided with the statement: “*Please write down any experiences of the chanting. This can include thoughts, feelings or body sensations that you may have noticed during or after the chanting*.”

#### Rating of Chanting Engagement

Participants rated their degree of chanting engagement, with responses ranging from 1 (*not at all*) to 5 (*I was silently repeating/chanting the sound the whole 12 min*), as a manipulation check to assess how much they followed the task instructions.

### Procedure

Participants were asked not to eat, drink, smoke or chew gum at least 40 min prior to testing in order to minimize saliva sample impurities as acidic or high sugar foods can compromise samples by lowering pH (Fukui & Toyoshima, 2013). All testing was done between 15:00 and 16:00 as these times have been recommended to control for circadian variations in cortisol levels, which are known to be most stable in the afternoon (Fukui & Toyoshima, 2013; Kirschbaum & Hellhammer, 1994; Pruessner et al., 1997). Participants were assigned to one of two conditions, vocal or silent chanting of the sound “om” for 12 min, guided by an audio recording. A consent form was completed followed by a saliva sample collection using a passive drool saliva collection aid (supplied by Salimetrics), to determine baselines levels of cortisol. Then the STAI and SRA (which included an additional 6 questions to assess cross-cultural altruism) were completed. Participants were then asked to maintain a straight spine whether sitting cross legged on the floor or on a chair and asked to either vocally or silently chant the sound “om” with a recording for 12 min. Following chanting, the STAI and SRA were completed again. Finally, participants also rated and wrote about their chanting experiences before providing another saliva sample.

### Analyses Approach

To determine the effects between before and after chanting, either vocally or silently, a series of 2 × 2 analyses of variance (ANOVA) with a between-subjects factor of Vocalisation (chanting vocally vs. chanting silently) and a within subjects factor of Time (before vs. after chanting) was conducted for each of the five measures. Interaction effects of ANOVAs were followed up by conducting a t-test on the difference in scores to assess the direction of the interaction.

The chanting experiences indicated by written reflections were assessed using a summative content analysis, which involves counting, and comparing keywords in qualitative descriptions (Hsieh & Shannon, 2005). This approach to qualitative data commences with searches of occurring words and calculating frequencies of each keyword. However, also considered, is the underlying meaning of content, going beyond merely counting words. For example, a summative analysis would classify words such as ‘sleepy’ and ‘drowsy’ into a single category of ‘tiredness.’ In the current study, words were tallied together for the vocal and silent chanting conditions, and then grouped into broader categories. These broader categories were then compared between the two chanting groups.

## Results

### Descriptive Statistics

Descriptive statistics for all measures before and after the chanting intervention (either vocally or silently chanting) are presented in Table 1.

**Table 1:** Participant’s scores before and after the chanting intervention

Condition	Measure	Before *M* (*SD*)	After *M* (*SD*)
Vocal	Cortisol (μg/dL)	0.15 (0.06)	0.13 (0.07)
	Anxiety(STAI-S)	38.38 (8.76)	28.94 (7.26)
	Altruism	50.88 (8.32)	52.63 (8.64)
	Cultural Altruism	23.5 (4.26)	23.62 (4.57)
Silent	Cortisol (μg/dL)	0.13 (0.07)	0.11 (0.05)
	Anxiety (STAI-S)	32.33 (8.83)​	27.06 (6.23)​
	Altruism	51.11 (8.55)​	53.17 (8.65)​
	Cultural Altruism	24.56 (3.57)​	25.06 (4.04)​

*Note.* Mean (*M*) and standard deviations (*SD*) (listed in parentheses) before and after chanting for both vocal and silent chanting measures of cortisol, stress, altruism and altruism toward other cultures (total n = 34)

### Analyses

#### Rating of Chanting Engagement

To determine if participants were engaged in chanting, a manipulation check was carried out that assessed the extent to which individuals engaged in the vocalisation or silent repetition of chanting throughout the experimental phase. The results of an independent t-test showed significantly higher scores in the vocal condition (*M* = 4.56, *SD* = 0.81) compared with the silent condition (*M* = 2.94, *SD* = 0.80). *p* < .001; *d* = 0.808, 95% CI [-2.18, -1.05]. This indicated that in the silent condition participants reported ‘mentally repeating the sound for about half of the 12 minutes’ as compared to the vocal condition where they reported to be ‘definitely chanting the sound for the whole 12 minutes.’

#### Physiological Stress (Cortisol)

**Fig. 1:**
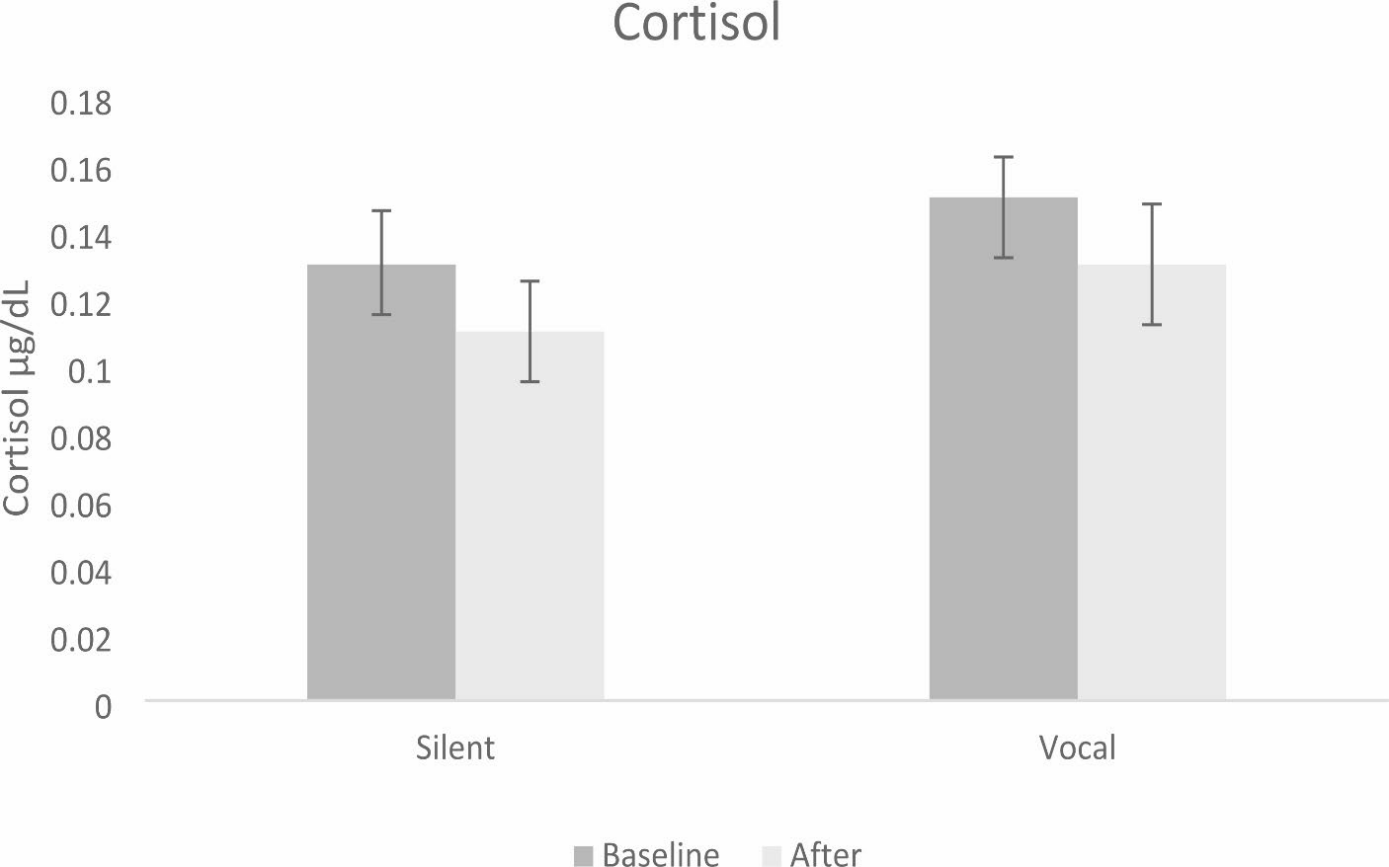
Cortisol Levels Before and After Vocal and Silent Chanting *Note*: Error bars represent ± 1 standard error of the mean (SEM)

A two-way mixed ANOVA showed a main effect of Time with cortisol decreasing significantly following chanting *F*(1,32) = 7.14, *p* = .012, $\eta _p^2$= 0.241. There was no significant effect of Vocalisation *F*(1,32) = 1.15, *p =* .292, $\eta _p^2$= 0.035 and no interaction *F*(1,32) = 0.02, *p =* .897, $\eta _p^2$= 0.001. Results are displayed in Fig. 1.

#### Anxiety (STAI-S)

**Fig. 2:**
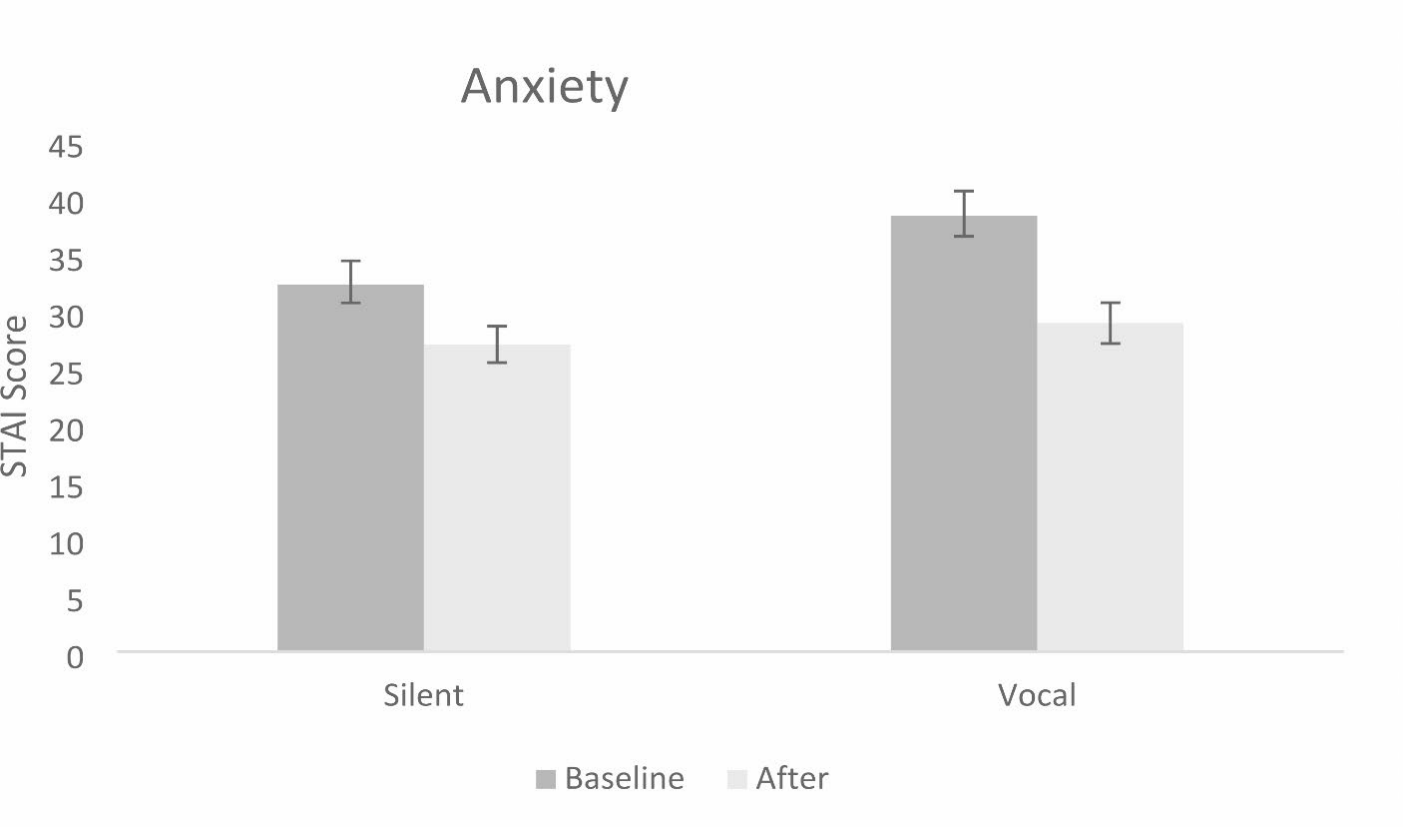
Self-Reported Anxiety Scores Before and After Vocal and Silent Chanting *Note*: Error bars represent ± 1 standard error of the mean (SEM)

A two-way mixed ANOVA showed a main effect of Time with anxiety decreasing significantly following chanting *F*(1,32) = 58.14, *p* < .001, $\eta _p^2$= 0.645. There was no significant effect of Vocalisation *F*(1,32) = 2.49, *p* = .125, $\eta _p^2$= 0.072, but there was a significant interaction between Time and Vocalisation, *F*(1,32) = 4.65, *p* = .039, $\eta _p^2$= 0.127. A post-hoc t-test on the change in scores revealed self-reported anxiety decreased more in the vocal condition (*M* = 9.44, *SD* = 7.05) compared to the silent condition (*M* = 5.28, *SD* = 3.94), *p* = .039, *d* = 0.73; 95% CI [0.23, 8.09]. Results are displayed in Fig. 2.

#### Altruism

**Fig. 3:**
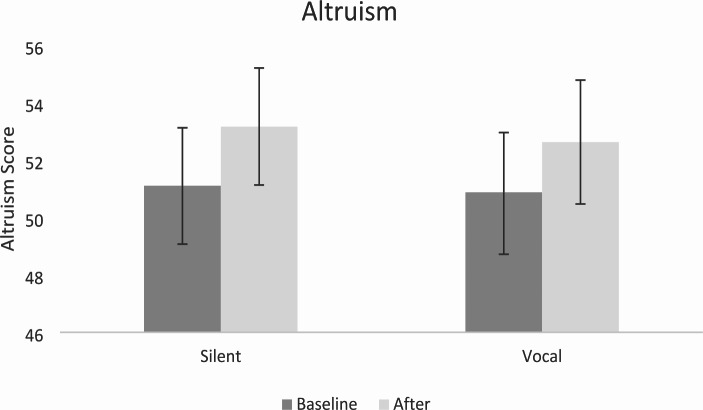
Altruism Scores Before and After Vocal and Silent Chanting *Note*: Error bars represent ± 1 standard error of the mean (SEM)

A two-way mixed ANOVA showed a main effect of Time, with altruism increasing significantly following chanting *F*(1,32) = 9.82, *p* = .004, $\eta _p^2$= 0.235. There was no effect of Vocalisation *F*(1,32) = 2.56, *p* = .893, $\eta _p^2$= 0.001 and no interaction *F*(1,32) = 0.40, *p* = .803, $\eta _p^2$= 0.002. Results are reported in Fig. 3.

A two-way mixed ANOVA of cross cultural altruism showed no main effect of Time *F*(1,32) = 1.00, *p* = .326, $\eta _p^2$= 0.030, no main effect of Vocalisation, *F*(1,32) = 0.81, *p* = .373, $\eta _p^2$= 0.025, and no interaction *F*(1,32) = 0.36, *p* = 553, $\eta _p^2$= 0.011.

#### Qualitative Measures

A summative content analysis was conducted on qualitative data to corroborate and extend quantitative measures and to further understand any differences between the phenomenology of vocal and silent chanting. Results of the summative analysis are reported in Table 2.

**Table 2:** Summary of frequency of words reported by participants describing their experience of vocal or silent chanting

Group	Words	Vocal (*N)*	Silent (*N)*
Sound	Vibration, vibrating, rhythm, voice, vocal, sound, listen, chant	53	36
Relaxed	Relax, relaxing, relaxed, calm, calmer, peace, peaceful, focus	37	38
Physical sensations	Breath, breathe, breathing, body, vibration, vibrating	19	19
Adverse effects	Worry, worried, worries, stress, stresses, stressed, anxious, uncomfortable, awkward	12	6
Mind	Mind, focus	12	8
Tiredness	Sleep, sleepy, sleeping, tired, drowsy	10	17

Participants in the vocal condition reported more words associated with sound such as ‘vibration’ and ‘rhythm’ than in the silent condition. For example, one participant reported, “I felt a vibration spread through the whole room as we chanted together” and another stated, “after a while I fell into a rhythm.”

Participants in the vocal condition reported more adverse effects such as feeling ‘worried’ or ‘uncomfortable.’ For example, one participant states that “hearing other people chant in the room was awkward at first.” and another writes, “I did feel a little uncomfortable occasionally as my voice started becoming hoarse and I couldn’t chant out the entire “om”.

Participants who vocally chanted also reported more words such as ‘mind’ and ‘focus’ compared with the silent condition. For example, one participant reported feeling “more focused” and another participant reported feeling “content with a clearer mind.”

Lastly, participants in the silent condition reported more words associated with tiredness such as ‘sleepy’ and ‘drowsy’ compared with those in the vocal condition. For example, one participant stated, “I could go to sleep right now” and another participant reports feeling “a bit drowsy.”

#### Additional Analysis

To check whether the removal of participants who could not provide saliva samples had any impact on results, we re-ran all analyses including these participants. We found no changes in significance of the results apart from anxiety where the interaction effect was no longer significant *F*(1,37) = 2.75, *p* = .106).

## Discussion

The current study set out to determine whether a brief intervention of vocal or silent group chanting would decrease stress, anxiety and increase altruism. Chanting was expected to decrease stress, anxiety, and increase altruism, with vocal chanting yielding stronger effects than silent chanting due to vocalization enhancing mechanisms such as slowed breathing, focused attention, synchrony, repetition, and rhythm. Results revealed that a short group chanting intervention, whether vocal or silent, decreased stress (cortisol levels), anxiety and increased altruism. Notably, self-reported anxiety reduction was more pronounced during vocal chanting compared to silent chanting. These results align with other religious and spiritual practices, such as conversational prayer and spiritual rituals, which have been found to promote solidarity in groups (Draper, 2014), as well as manage distress (Krause et al., 2000), and negative emotions (Sharp, 2010). Collectively, these findings provide evidence for the potential of both vocal and silent chanting practices in reducing stress and fostering social connection.

### Physiological Stress

Consistent with predictions, both vocal and silent chanting resulted in decreased cortisol levels, aligning with previous research on cortisol reduction following singing and music listening (Fukui & Toyoshima, 2014; Kreutz, 2014; Kreutz et al., 2004). However, no significant difference was observed between the two chanting conditions. The discrepancy between our results and studies indicating a greater decrease in cortisol levels following singing compared to music listening (Kuhn, 2002; Unwin et al., 2002), suggests the involvement of various factors influencing the cortisol response. One potential explanation concerns the level of attention and engagement required by the chanting conditions investigated. Silent chanting may require a higher level of focus and active engagement compared to passive music listening, potentially influencing cortisol reduction. Another factor that may contribute to the mixed findings is the duration of the chanting intervention. Our study utilised a relatively short intervention of 12-minutes, which closely resembled the 5-minute intervention utilized by Fukui and Toyoshima (2014). In contrast, Kuhn (2002) employed longer interventions of 30 min involving either singing or music listening. It is plausible that an extended duration of chanting or music engagement triggers more pronounced physiological responses, leading to additional decreases in cortisol levels.

### Anxiety

As anticipated, self-reported anxiety levels decreased following both vocal and silent chanting interventions. These findings align with the study conducted by Wolf and Abell (2003), which demonstrated the stress-reducing effects of chanting and its impact on depressive symptoms. A possible mechanism underlying the reduced anxiety observed in both chanting conditions is the enhanced cognitive and emotional capacities resulting from focused attention. By directing attention to the sounds and rhythm of chanting and disengaging from habitual or automatic thinking, individuals may experience increased emotional stability and cognitive control, ultimately leading to a sense of relaxation (Chiesa, 2013). This focused attention may also contribute to the reduction of intrusive thoughts, which are associated with increased stress (Barnhofer et al., 2010). Taken together, these findings suggest that chanting, regardless of whether it is performed vocally or silently, is associated with decreased self-reported anxiety levels. The cognitive and emotional engagement fostered by focusing attention on chanting may contribute to anxiety-reducing effects observed in the current study.

Consistent with our predictions, self-reported anxiety showed a greater decrease in the vocal chanting condition. This finding has important theoretical implications regarding the specific mechanisms through which vocal chanting may lead to a greater reduction in anxiety compared to silent chanting. Several factors may contribute to the greater reduction in self-reported anxiety observed in vocal chanting.

One possible explanation for greater anxiety reduction observed in vocal chanting is the direct impact on respiration. Vocal chanting involves explicit vocalization of sounds, which can influence breathing patterns (Bernardi et al., 2000, 2001a, b), potentially inducing feelings of relaxation (Jerath et al., 2006). In contrast, silent chanting may not yield the same explicit influence on breathing as vocal chanting. The direct physical engagement in vocal chanting may enhance anxiety-reducing effects. However, further research is required to determine the exact differences between respiration rates in vocal chanting compared to silent chanting. Although Kreutz et al. (2004) and Kuhn et al. (2002) found music engagement to reduce stress more than music listening, breathing rate was not measured. It is possible that listening to chanting, as in the silent condition, could also impact breathing. Future research could compare breathing rates of vocal and silent chanting to uncover underlying mechanisms.

Another factor that may contribute to the greater anxiety reduction in vocal chanting is the potential improvement in concentration and focus. Indeed, participants in the vocal condition expressed a higher frequency of words such as ‘focus’ indicating higher levels of concentration. In contrast, participants in the silent condition expressed a higher frequency of words associated with tiredness, indicating lower levels of focus. Silent chanting may be more prone to lapses in concentration, allowing the mind to wander, potentially diminishing its impact on anxiety. This is supported by research that has found people are less happy when their minds are wandering (Killingsworth & Gilbert, 2010). Furthermore, research has found reduced anxiety and emotional stress when participants use attentional control techniques (Brefczynski-Lewis et al., 2007).

Lastly, engagement in the vocal chanting condition may be more synchronous and consistent creating a more immersive and cohesive experience compared to silent chanting. This is supported by the manipulation check with participants in the vocal condition reporting to have engaged significantly more than those in the silent condition. This could have a more profound effect on the psychophysiological state, contributing to greater reductions in self-reported anxiety. This also aligns with research by Unwin et al. (2002), which demonstrated more pronounced effects of singing compared to passive listening on positive mood. Future research could investigate whether chanting engagement moderates or mediates psychological or physiological benefits of chanting with a larger sample size.

Taken together, several factors could be involved with results indicating that anxiety reduced more in vocal chanting compared to silent chanting, however, more research is needed to specifically investigate the underlying mechanisms involved. Moreover, while initial findings showed greater anxiety reduction in the vocal condition for participants with complete datasets, this interaction effect was negated when including the additional 5 participants who couldn’t provide saliva samples. This highlights the necessity for further research to thoroughly investigate whether there are indeed any advantages of vocal chanting compared to silent chanting.

### Social Connection

As expected, both vocal and silent chanting were found to increase self-reported altruism, aligning with previous research on social connection that demonstrates synchronous activities facilitate cooperation (Kokal et al., 2011; Valdesolo et al., 2010; Wiltermuth & Heath, 2009). In particular, singing in groups has been found to increase group cooperation, strengthen social bonds, and this has even been suggested to be a key function of group singing (Tarr et al., 2016; Wiltermuth & Heath, 2009). Moreover, physiological markers such as oxytocin, related to social connection have been found to increase from singing in groups (Good & Russo, 2022). Interestingly, altruism was not influenced by vocalization, in contrast to Perry et al. (2016) who found vocal chanting resulted in a greater rise in altruism compared to silent chanting among novice meditators. There are a few possibilities as to why altruism may have increased in both conditions.

Firstly, silent chanting enabled participants to mentally simulate vocalization. Particularly, mentally simulating sounds with the recording may have fostered a sense of group participation and a shared goal. Secondly, the lack of vocalisation in the silent condition may have promoted deep relaxation and group comfort. Indeed, qualitative responses indicate participants felt “worried” and “uncomfortable” twice as more in the vocal condition compared to the silent condition. Therefore, while the vocal condition provided an opportunity for explicit synchronisation, the silent condition may have nurtured a sense of connection through a relaxed communal atmosphere and shared objective.

Contrary to predictions, the current study provided no evidence of increased altruism toward other cultures following either vocal or silent chanting. It is plausible that the potential impact of chanting on altruism toward other cultures was inhibited by a ceiling effect, given that scores were already high at baseline in both the vocal (M = 23.5, SD = 4.26) and silent (M = 24.56, SD = 3.57) chanting groups. Consequently, there might have been limited possibility for this type of altruism to increase beyond baseline levels. Another possibility is that items created for this study to measure altruism toward other cultures were not sufficiently sensitive to detect types of changes we were looking for, or perhaps chanting does not affect this type of altruism. Further refinement of measurement tools and control groups may be necessary to adequately capture altruism toward other cultures as well as including valid measures of social connectedness and a behavioural measure of altruism. Taken together with previous findings, effects of vocal and silent chanting on altruism may vary and further research is needed to disentangle mechanisms that contribute to observed differences.

### Phenomenology of Chanting

This study also included a qualitative component, utilizing a summative content analysis to complement quantitative measures and gain further insights into distinctive effects between vocal and silent chanting. Analysis of the qualitative data revealed notable differences in the phenomenology experienced by participants in each condition. This suggests differences in social dynamics between vocal and silent chanting. Participants who engaged in vocal chanting reported a higher frequency of words associated with sound, such as ‘vibration’ and ‘rhythm,‘ indicating more sensory engagement during the practice. This finding is logical due to the explicit vocalisation in the vocal chanting condition which likely also helped to maintain regular rhythm with the group.

Participants in the vocal condition also reported adverse effects, including feelings of discomfort or worry, which could be attributed to shyness, embarrassment, or performance anxiety. Performance anxiety is associated with feelings of being evaluated or observed by others, so this may have influenced participants in the current study as they were novice chanters and perhaps not used to singing in a group. Further, performance anxiety is known to be linked to social and general anxiety (Dobos et al., 2019; Henshaw & Collyer, 2022; Kokotsaki & Davidson, 2003). This is supported by one participant stating, “hearing other people chant in the room was awkward at first” and another who expresses being self-consciousness by reporting “I did feel a little uncomfortable occasionally as my voice started becoming hoarse.” Taken together, these findings indicate participants may be a little uncomfortable in the vocal condition due to the novel experience of chanting in a group.

Participants in the vocal condition expressed a higher frequency of words such as ‘mind’ and ‘focus’, suggesting increased cognitive engagement during chanting. This finding aligns with quantitative results showing a greater reduction in psychological stress in the vocal condition compared to the silent condition, suggesting participants’ increased engagement contributed to a more pronounced psychological stress-reducing effect. Conversely, participants in the silent condition exhibited a higher frequency of words associated with tiredness, such as ‘sleepy’ and ‘drowsy,‘ indicating a potential relaxation effect and lack of focus. Lastly, frequency of words such as ‘relaxation,’ ‘calm,’ and ‘breathing’ were similar in both vocal and silent conditions, suggesting both conditions had a calming effect. This finding supports quantitative data revealing decreased cortisol levels in both conditions. Taken together, the qualitative data obtained from participants’ descriptions align with and support the quantitative results of this study, providing a more comprehensive understanding of the psychological and physiological effects of vocal and silent chanting.

### Limitations

The present study provides a foundation for future investigations into the effects of chanting on stress and altruism. Although the present study did not include a non-chanting (completely silent) control group, baseline measures served as a within-subjects control for our two experimental conditions. Future investigations would benefit from the inclusion of a non-chanting control group to determine if observed differences in anxiety were attributable to different styles of chanting or to a higher level of engagement in the vocal condition. Another limitation of this study pertains to the cortisol measurements, which were only taken at two time points. Cortisol levels are known to fluctuate over time and so a more effective procedure would be to obtain cortisol measures at multiple timepoints during the day before and after chanting. Another option would be to have longer chanting sessions which may have led to greater impact. Assessing cortisol levels at multiple time points and having longer sessions may provide a clearer picture of the sustained effects of chanting. Future research could also measure differences in respiration rate between vocal and silent chanting to see if this was something that contributed to decreased self-reported stress in the vocal condition. This would determine if vocalising is necessary at all or whether benefits can be obtained with silent chanting.

Another limitation of the current study is that demographic data such as culture, ethnicity, socioeconomic status or religiosity was not collected. This makes it difficult to determine whether the contact hypothesis (that intergroup contact may reduce prejudice) was relevant as it is unknown how many different cultures were in groups together. Therefore, it is unclear whether the null findings of altruism toward other cultures were due to small non-diverse chanting groups or something else. Future research could examine more diverse and larger chanting groups with validated and reliable measures of cross-cultural altruism. Furthermore, past research has found participants who report high spirituality, also report enhanced effects of chanting (Perry et al., 2021). In another study on how religion impacts inequities in stress and health, DeAngelis et al. (2023) found higher religious and spiritual beliefs were linked to higher resiliency biomarkers for Black women but lower levels for White respondents. These results suggest it may be important to include religious beliefs and ethnicity in future research in the case that such diversity impacts how individuals respond to chanting. Addressing limitations and expanding this research in future would enhance the validity and depth of the current findings, contributing to a more thorough understanding of physiological and psychological benefits of chanting, as well as how chanting impacts different groups of people.

The current investigation highlights the potential of chanting to reduce stress, foster social bonds, and promote cooperation. The development of novel stress reduction methods and strategies to promote cooperation is essential, given the escalating prevalence and associated costs of heightened stress and social disconnection (Hawthorne, 2008; Lee et al., 2020; Okruszek et al., 2020). Group chanting may hold potential to benefit individuals while also positively impacting society. Chanting provides significant physiological and psychological benefits enhancing social connection and reducing stress and anxiety. Further research is needed to elucidate the underlying mechanisms of such benefits, along with the long-term effects of chanting, and to explore ways that chanting may foster altruism towards people from different cultures. Such research would have practical applications contributing to individuals leading healthier, more balanced lives as well as enhancing social cohesion.

## Data Availability

The data presented in this study are openly available in [OSF] at 10.17605/OSF.IO/FGVZ3.
